# Polymalic acid for translational nanomedicine

**DOI:** 10.1186/s12951-022-01497-4

**Published:** 2022-06-21

**Authors:** Xing Huang, Liusheng Xu, Hui Qian, Xinghuan Wang, Zhimin Tao

**Affiliations:** 1 Center for Evidence-Based and Translational Medicine, Department of Urology, Zhongnan Hospital of Wuhan University, Wuhan, 430071 Hubei China; 2 Jiangsu Key Laboratory of Medical Science and Laboratory Medicine, School of Medicine, Jiangsu University, Zhenjiang, 212013 Jiangsu China; 3 Zhenjiang Key Laboratory of High Technology Research On Exosomes Foundation and Transformation Application, School of Medicine, Jiangsu University, Zhenjiang, 212013 Jiangsu China

**Keywords:** Synthesis, Bioproduction, Nanoscale, Disease diagnosis, Therapy

## Abstract

With rich carboxyl groups in the side chain, biodegradable polymalic acid (PMLA) is an ideal delivery platform for multifunctional purposes, including imaging diagnosis and targeting therapy. This polymeric material can be obtained via chemical synthesis, or biological production where L-malic acids are polymerized in the presence of PMLA synthetase inside a variety of microorganisms. Fermentative methods have been employed to produce PMLAs from biological sources, and analytical assessments have been established to characterize this natural biopolymer. Further functionalized, PMLA serves as a versatile carrier of pharmaceutically active molecules at nano scale. In this review, we first delineate biosynthesis of PMLA in different microorganisms and compare with its chemical synthesis. We then introduce the biodegradation mechanism PMLA, its upscaled bioproduction together with characterization. After discussing advantages and disadvantages of PMLA as a suitable delivery carrier, and strategies used to functionalize PMLA for disease diagnosis and therapy, we finally summarize the current challenges in the biomedical applications of PMLA and envisage the future role of PMLA in clinical nanomedicine.

**Graphical Abstract:**

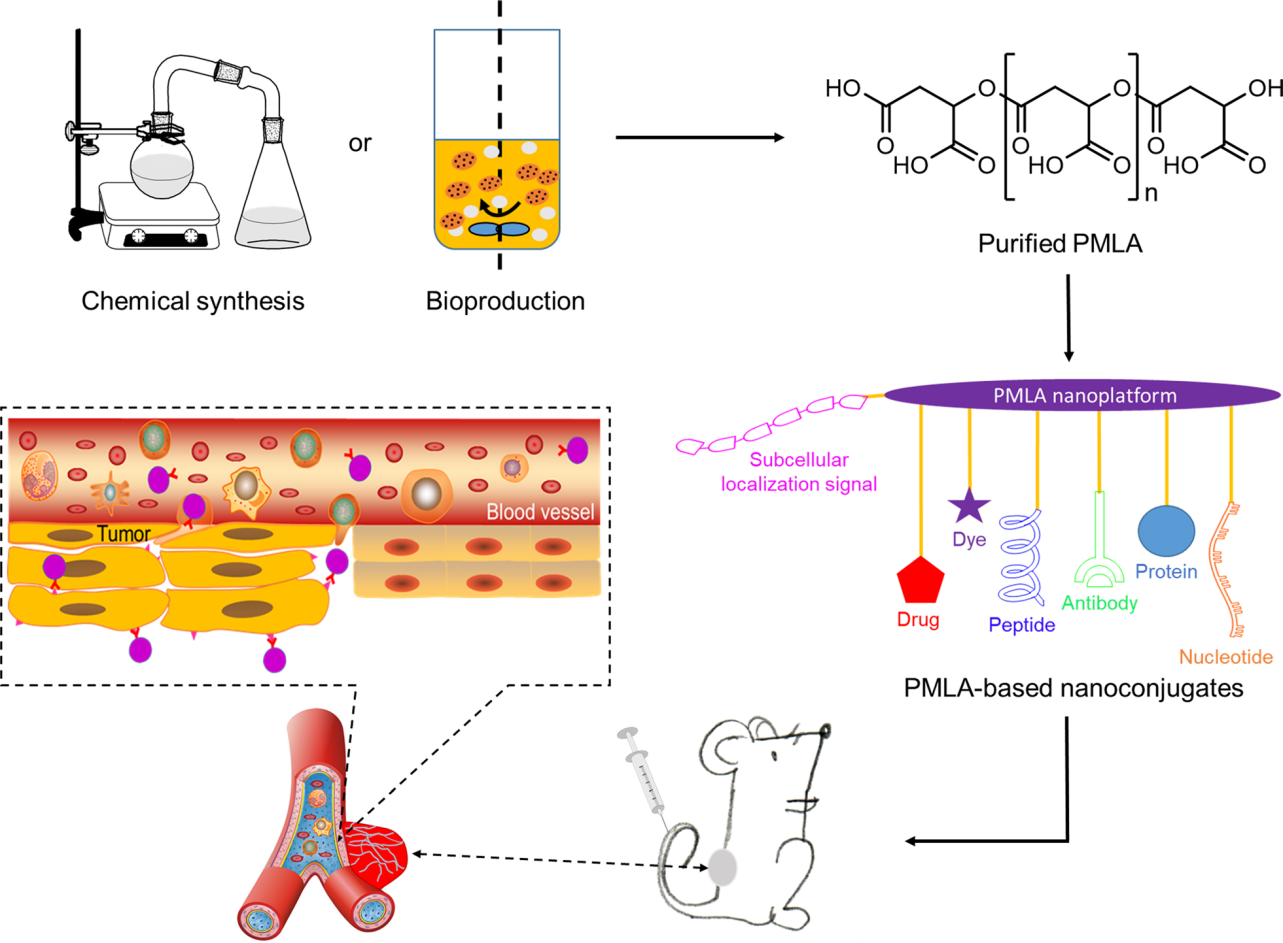

## Highlights

- The biosynthesis of polymalic acid (PMLA) and its biotechnical high-grade production from microorganisms compared with the chemical synthesis of PMLA
- The physicochemical and biological characteristics of PMLA and its derivatives
- How PMLA’s general chemical characteristics can be used to generate various macromolecular compounds for pharmaceutical delivery
- The concepts of biological and clinical targeting exemplified by PMLA-based drugs and imaging agents and their biodistribution and biodegradability
- An evaluation of the mechanisms that generate preclinical antitumor efficacy and the translational potential for clinical imaging

## Introduction

**Fig. 1:**
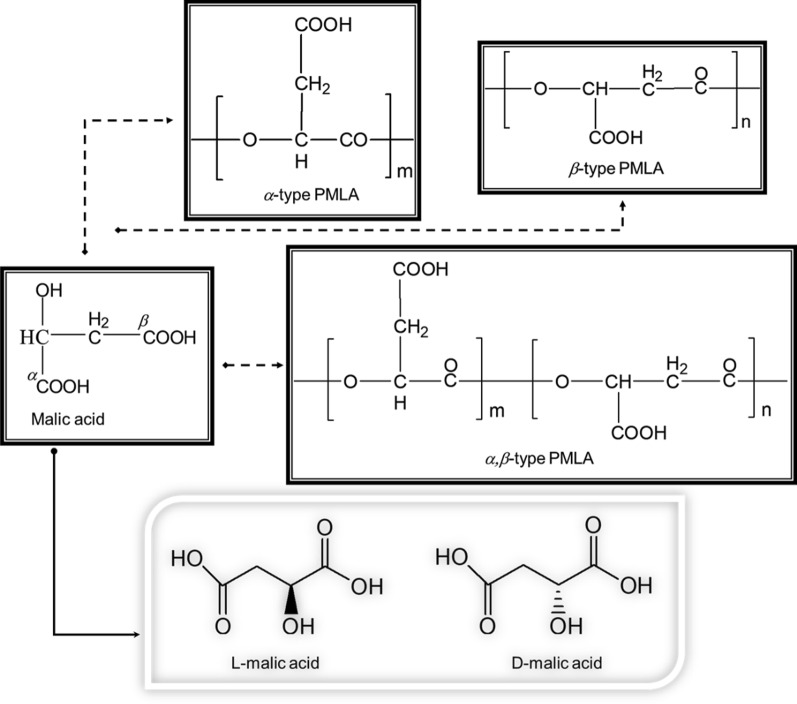
Structures of PMLAs, including structures of α-, β-, or α, β-types of polymalic acid and malic acid in its l- or d-form

Poly(β-l-malic acid) (β-PMLA) is a polymeric material formed by ester bonds between the hydroxyl and β-carboxyl groups of l-malate [1, 2]. Only β-PMLA is naturally available when produced by certain microorganisms, mainly *Aureobasidium pullulans* (*A. pullulans*) and *Physarum polycephalum* (*P. polycephalum*), whereas all α-, β-, and α, β-PMLAs can be chemically synthesized (Fig. 1). Although malic acid conforms in both d- and l-types, l-malic acid, the sole monomer of PMLA, exists as an intermediate metabolite in the tricarboxylic acid (TCA) cycle [3]. Because PMLA finally decomposes into the unique hydrolysates of l-malate, it possesses minimal immunogenicity or cytotoxicity while still maintaining full biodegradability and biocompatibility [4]. In addition, given the high density of carboxyl groups in its side chains (~ 862 free carboxylates per PMLA of a molecular weight *M*_*w*_ = 100 kDa) (Fig. 1), PMLA is highly water-soluble and chemically reactive, making it ideal for further pharmaceutical attachment.

Polymalic acid was first isolated from the secretions of *Penicillium cyclopium* in 1969 [5] and has been examined for its biochemical roles in protein homeostasis and nucleic acid synthesis [6, 7]. PMLAs were not chemically synthesized until benzyl malolactonate was polymerized to form PMLA of *M*_*w*_ < 6 kDa in 1979, thereby considered as a potential drug carrier for the first time [8]. With the rapid development in bioresource technology, research efforts on the bioproduction of PMLAs with relatively large *M*_*w*_ have intensified [1, 9]. Using economic fermentation, PMLA biosynthesis is taking steps toward a large-scale and continuous standardized production [9]. Lately, the search for polymalatase that is responsible for PMLA biosynthesis has shown exciting results, especially with the input of the CRISPR/Cas9 gene editing technique [10, 11]. Those advances would lead to the microbial biotechnology to manufacture PMLA in a controlled manner and at an industrial scale, thus imparting PMLA with sustainability and renewability, eventually superior to its chemical synthesis.

Chemistry-wise, the side-chain carboxyl groups of PMLA allow their simple and effective coupling with many functional groups in biologically active molecules, including targeting ligands, chemotherapy drugs, imaging agents, and therapeutic antibodies, to construct a variety of PMLA derivatives with preserved hydrophilicity [4, 12]. Furthermore, PMLA can be fully metabolized, recycled via the mitochondrial TCA cycle, and reused by cellular energetics [13]. The modification of PMLA into its functional derivatives using PMLA as a working platform enables the significant improvement of drug bioavailability to diseased regions, making PMLA successful vehicles for pharmaceutical delivery. The unrivaled features of PMLA have made it an excellent biomaterial for medical packaging and pharmaceutical delivery.

In this review, we first explore an overview of the chemical and biological synthesis of PMLA, emphasizing the recent research findings to understand the PMLA biosynthetic pathway. We next summarize the physicochemical and biological characteristics of PMLA as a polymeric delivery platform at nanoscale and elaborate on the versatile modification of diverse functional groups to enhance disease diagnosis and treatment. Finally, based on the chemistry via which the PMLA-based materials have been finalized, we revisit the current biomedical applications of PMLA in diverse diseases and envision its future role in translational and clinical medicine.

## Chemical synthesis of PMLA

**Fig. 2:**
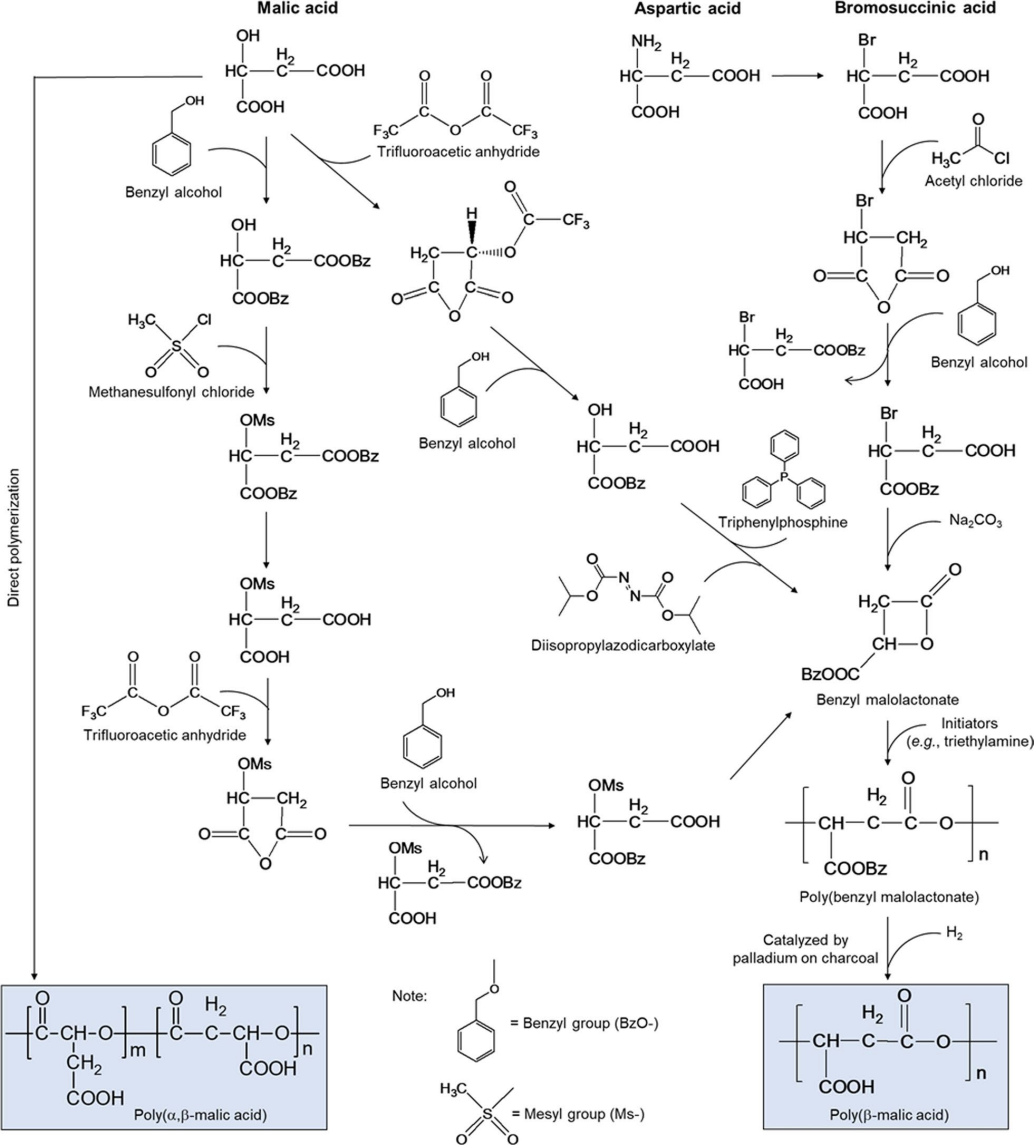
Different chemical syntheses from malic acid, aspartic acid, or bromosuccinic acid to PMLA [14, 15]

The chemical routes to synthesize PMLAs and their derivatives have been intensively studied, beginning with the homopolymerization or copolymerization of different monomers and ending up with enantiomeric or racemic mixtures of PMLAs (Fig. 2) [14, 15]. These syntheses are categorized into two types of chemical reactions: direct polycondensation and ring-opening polymerization.

### Direct polycondensation

Direct polycondensation of l-malic acid, in the presence of catalysts such as tin(II) chloride, generates α, β-PMLA with the liberation of water via a one-step reaction [15, 16]. This method requires simple synthetic conditions, including few organic solvents (e.g., diphenyl ether) employed and reaction heating to 110–140 °C (going beyond 140 °C may convert malic acid into fumaric acid), but yields a high purity and large quantity of PMLA (as high as > 99%), suitable for industrial scaleup. The molecular weight of the products can be tailored as well by varying the reaction time, temperature, and the choice of catalyst; however, many side reactions (e.g., transesterification) may occur and the by-products (e.g., malic acid oligomers) are difficult to separate from the synthesized PMLA. Furthermore, because every malic acid molecule contains only one -OH but two -COOH groups, which limits the condensation reaction, and every water molecule generated from the reaction cumulatively undermines the condensation, this method can only yield PMLAs of *M*_*w*_ < 6 kDa. This low *M*_*w*_ product renders this method of production impracticable for biomedical purposes. To overcome these problems, hydroxyl-rich comonomers (e.g., β-cyclodextrin) have been recently employed to construct malic acid–based polyesters via direct condensation with techniques to remove water in a timely fashion [17, 18].

### Ring-open polymerization

Alternatively, ring-opening polymerization can be further differentiated into cross-ester ring-opening polymerization and lactone ring-opening polymerization, based on the type of monomers used as the initial reactants. In a typical synthesis of PMLA via cross-ester ring-opening polymerization, β-benzyl l-malate is prepared by using l-malic acid or l-aspartic acid as the precursor. Malide dibenzyl ester is an intermediate reaction product resulting from the intermolecular dehydration of two molecules of β-benzyl l-malate in the presence of ZnO catalyst, followed by a ring-opening homopolymerization at 220 °C for 8–10 h and a debenzylation reaction with the addition of a palladium-carbon catalyst [19]. As a result, α-PMLA of *M*_*w*_ = 1.5–4 kDa is synthesized with a low yield, because the polymerization activity of cyclic malide dibenzyl ester remains very small; henceforth, this cross-ester method of ring-opening polymerization to synthesize PMLA is not scalable.

Lactone ring-opening polymerization begins with β-substituted β-lactone (e.g., benzyl β-malolactonate) in monomeric form that reacts with initiators, such as triethylamine, to yield the benzyl ester of β-PMLA that is further hydrogenated using palladium on charcoal as a catalyst. In this reaction, monomeric benzyl β-malolactonate is synthesized from malic, aspartic, or bromosuccinic acid (Fig. 2) [14]; for example, using malic acid as the starter, a methylsulfonyl group (Ms) or trifluoroacetic anhydride is introduced into the reaction in order to replace the active hydrogen of malic acid’s hydroxyl group. The carboxyl groups of malic acid are protected by benzylation, which proceed through stepwise reactions to form benzyl malolactonate for the continued ring-opening polymerization of β-PMLA (*M*_*w*_ = 7–20 kDa) [20, 21]. Similarly, using aspartic acid (or converting it to bromosuccinic acid) as a starter initiates a reaction with acetyl chloride to form bromosuccinic anhydride. Subsequent benzylation in which only the β-brominated derivatives can cyclize in the presence of sodium carbonate to obtain benzyl β-malolactonate, finally yields an optically active β-PMLA of *M*_*w*_ up to 150 kDa through catalytic hydrogenolysis [22–25]. The ring opening of benzyl β-malolactonate for further polymerization creates a new opportunity to synthesize PMLA of high *M*_*w*_ and becomes a leading synthetic method to acquire high-yield PMLAs and their derivatives [26–28].

### Limitations of chemically synthetic PMLA in pharmaceutical applications

Synthetic PMLAs from the aforementioned chemical reactions have been evaluated for biomedical applications. For instance, PMLAs have been used as fusogenic materials with artificial phospholipid bilayers (e.g., liposomes) to enhance their thermo- or pH- sensitivities in medicinal uses [29, 30]. However, as discussed above, the chemical synthesis of PMLA requires harsh reaction conditions such as the use of high temperatures, vacuums, and precious but noxious catalysts. These harsh reaction conditions are neither ecologically nor economically friendly, thereby preventing the widespread use of synthetic PMLA in compendious biological applications. At the same time, the yield of chemical synthesis is relatively low and unsuitable for further industrial scaleup. Moreover, synthetic PMLA cannot obviate the racemization of different enantiomers, and the possible contamination of D-malic acid with unknown biological fate may have a potential health risk. The chemical synthesis of PMLA has been well documented in the literature [14, 31]; however, research on overcoming these obstacles in the chemical synthesis of PMLA has been fairly stagnant over the past decade.

## Biosynthesis of PMLA

Biological routes using natural microorganisms under optimized culture conditions can produce poly(β-l-malic acid) from sustainable sources through one-pot fermentation, revealing the engineerable potential of biosynthetic PMLAs [32, 33]. In contrast to chemically synthetic PMLAs, biosynthetic PMLAs are β-type linear polymers made up of only l-malate units, i.e., poly(β-l-malic acid)s, providing many structural advantages over the other two types (i.e., α-, and α, β-PMLA). On one hand, the β-type PMLA contains a four-atom backbone rather than a three-atom backbone as in the α-type, with an expansive full-length polymer chain that maximizes its accessibility to the functional groups to be attached [34]. On the other hand, there is five-atom space between the two adjacent carboxylic acid pedants in the β-type PMLA. This specific spacing optimizes the physicochemical parameters of the side-chain substituents, including their individual hydrophobicity, charge neutralization capacity, ligand length, and density, thus forming an energy-minimized structure with a favorable molecular geometry and charge distribution for its effective interplay with biological membranes [35]. In the following text, PMLA only refers to biogenic poly(β-l-malic acid) unless otherwise specified.

**Fig. 3:**
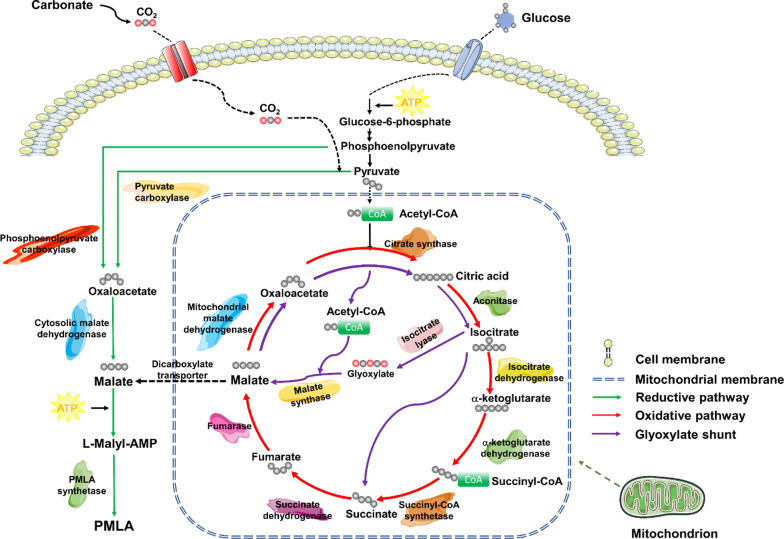
Schematic illustration of generic biosynthetic pathways of PMLA in microorganisms. Using glucose as carbon source, it is converted into phosphoenolpyruvate and pyruvate by glycolysis, both finally producing malate in the cytoplasm via the reductive pathway. In parallel, pyruvate is transported into the mitochondria, is oxidized and decarboxylated to form acetyl-CoA, and then joins the TCA cycle. After acetyl-CoA condenses with oxaloacetate to form citric acid, it undergoes a series of enzymatic reactions to produce malate via the oxidative pathway. Alternatively, a shortcut, the glyoxylate shunt, utilizing glyoxylate as an intermediate can bypass the TCA cycle to produce malates. Malates produced inside mitochondria are transported to the cytoplasm through a dicarboxylate transporter, where PMLA synthetase finally polymerizes them into PMLA

Being a direct precursor of PMLA, malic acid can be produced via three metabolic pathways: the reductive pathway in the cytoplasm, the oxidative pathway including the TCA cycle and the glyoxylate shunt inside the mitochondrial matrix, in which exogenous carbonate serves as a shifter (Fig. 3) [13]. The metabolic balance between intra- and extra- mitochondrial malate production is maintained by the malate–aspartate shuttle as overproduced oxaloacetate is transported into the mitochondria and a dicarboxylic acid carrier transfers excess malate from the mitochondria to the cytosol [36, 37]. Malate is only polymerized into PMLA outside the mitochondria [38, 39]. There have been research efforts in search of PMLA synthetase for over two decades. Although the enzymatic synthesis of PMLA in microorganisms is not fully understood, mounting evidence has revealed a generic synthesis of PMLA in myxomycetes (e.g., *P. polycephalum*) and fungi (e.g., *A. pullulans*) through the machinery of nonribosomal peptide synthetase (NRPS) (Fig. 3) [40]. The putative PMLA polymerase may embrace dual active sites, possessing enzymatic functions of both malyl ligase and malyl polymerase, whereas the former activates the malyl-AMP (*P. polycephalum*-specific) or malyl-CoA (*A. pullulans*-specific) ligation, and the latter unites a malyl-ligand to the other malate, forming dimer, oligomers, and polymers of different lengths [9, 39].

In *P. polycephalum*, PMLA is only synthesized at the plasmodium stage in the nuclei of multinucleate coenocytes. PMLA has not been found in any of its mononucleate forms in the life cycle of *P. polycephalum*; however, the synthetic rate of PMLA production increases in parallel to mitotic nuclei enlargement, evidencing the indispensable role of PMLA in maintaining synchronous functioning [41]. Structurally resembling the backbone of DNA, PMLA binds DNA polymerases and nuclear proteins (e.g., histones), actively coordinating nucleic acid synthesis and protein homeostasis [42, 43]. To maintain a constant level in the nucleus, endogenous PMLA in excess is shuttled to the cytoplasm, while exogenous PMLA via microinjection into plasmodial veins is effectively relocated into the nuclei that are saturated with intrinsic PMLA, leading to a substantially shortened cell cycle and an augmented growth rate [41, 44]. A temporary leakage in the plasmodial membrane may disrupt PMLA synthesis to some extent, but cell lysates of *P. polycephalum* plasmodia completely block the synthetic activity of malyl polymerase (but not malyl ligase), suggesting a tyrosine kinase–dependent cell injury signaling in deactivating the unidentified PMLA synthetase[39]. In addition, a plasmodium-specific polypeptide spherulin 3b (11.3 kDa, also named NKA48) assists in the PMLA synthesis, as the knockdown of NKA48 mRNA significantly lowers PMLA concentration [45]. Nonetheless, the identification of PMLA synthetase in *P. polycephalum* has been unsuccessful, partially because cell extracts are void of synthetase activity, and purified PMLA from *P. polycephalum* usually has a *M*_*w*_ of 50–300 kDa [8].

Research on PMLA production using yeastlike fungi is ongoing [1, 9, 46]. In contrast to myxomycetes, fungi (e.g., *A. pullulans*, *Aureobasidium* spp.) produce high yields of PMLAs throughout their life cycles (except hyphae), with lower *M*_*w*_ of < 200 kDa, more branched structure, and less polydispersity [46–48]. Building a genome-wide metabolic model of one *A. pullulans* strain, researchers acquired and cloned a specific ligase in the conversion of malate to malyl-CoA before the polymerization of malyl-CoA into PMLA by catalysis with an unknown enzyme [49]. Recently, a newly identified PMLA synthetase gene in *A. melanogenum* has been reported, responsible for transforming malate into malyl-AMP as well as linking malyl-AMP to PMLA [11]. This gene segment contains 5049 base pairs, encoding a typical NRPS, which includes an adenylation domain binding ATP (and forming malyl-AMP); a thiolation domain with a 4′-phosphopantetheine arm, which can bind a malyl-enzyme while releasing AMP; and a condensation domain, which can esterify two malate ligates into one dimer [50, 51]. This gene expression is turned on when the transcription factor Crz1 is translocated into the nucleus, induced by an influx of Ca^2+^. Regulation of the gene’s expression occurs when Ca^2+^ becomes unavailable, which drives Crz1 into the cytoplasm and thus turns off the gene expression and stops the production of PMLA [11]. Although it is yet to be isolated, this PMLA synthetase is thought to sit across the plasma membrane with six transmembrane domains in its N-terminal, where no thioesterase domain is revealed as one essential unit in a typical NRPS for catalyzing the dissociation of the newly made PMLA from the synthetase [50]. More work needs to be done before this PMLA synthetase can be fully characterized and understood.

## Biodegradation of PMLA

Polymalic acid hydrolases have been discovered in a wide range of eukaryotes and prokaryotes, including bacteria that produce no PMLA but some of them adopt PMLA as carbon source for growth [47, 52]. A 68-kDa PMLA hydrolase (also known as depolymerase or polymalatase) was first isolated from the cytosol and culture medium of *P. polycephalum*, and it revealed the specific removal of malate from PMLA [53]. Different from typical poly(β-hydroalkanoate) depolymerases, PMLA hydrolase resembles glucosidase and exhibits no metallo- or serine-esterase activity with its peak catalysis at pH = 3.5, and represents an extracellular glycoprotein that has minimal activity intracellularly [53]; therefore, being devoid of hydrolytic activity at pH =  ~ 6.5 in the cytoplasm, PMLA hydrolase stationed at the entrance of the nuclear pore (never entering the nucleus) and serves in vivo as a molecular chaperone to transport PMLA into and out of the nucleus. With one polymalatase zymogen (200 kDa), two forms of polymalatase (68 kDa), and one glycosylated polymalatase (97 kDa), the proteolytic fragments of PMLA hydrolase may coexist [38, 54]. There is a large excess of PMLA versus polymalatase in the cytoplasm, and as intranuclear PMLA remains constant for physiological maintenance, the surplus of PMLA in the cytoplasm is delivered by polymalatase. This is accomplished via exocytosis, when the hydrolase activity is triggered by a plasma membrane-bound tyrosine kinase and a fraction of the polymalatase is secreted into the culture medium together with PMLA [54, 55]. Enzymatic degradation then begins with the hydroxyl end of PMLA and proceeds to the carboxyl terminal through a specific binding to the 2nd malyl residue from the hydroxyl end and an electrostatic binding to the 12th malyl residue down on the polymeric chain for steric stabilization [56]. This depolymerization by PMLA hydrolase can be halted by the following variations: (1) the d-enantiomer is mingled in the malyl unit; (2) fungal PMLA is used as substrate, with probable branched structures and covalent bonds with polysaccharides; (3) a hydrophobic substitution on the pendant α-carboxylate may restrain the substrate from polymalatase binding; and (4) β-carboxyl groups become terminally capped dimers or cyclic PMLAs [57, 58].

The exact physiological role of PMLA in fungi is not fully understood yet. Research has, however, uncovered several facts about PMLA from fungal origin. First, the complete removal of the putative PMLA synthetase gene from the fungal genome was found to have no effect on cell growth, and the full restoration of the synthetase gene only recovered one-third of the original PMLA production [11]. Second, fungal PMLA was found only embedded in cell membranes and was slowly released into the culture medium; it barely inhibited DNA polymerase α and was only partially soluble in acetone, thus showing a distinctive feature from the one with *P. polycephalum* origin [47]. To date, there are not many studies regarding the intracellular localization and relocation of polymalatase in fungi. In contrast, prokaryotic PMLA hydrolase has been isolated from several bacteria, which employ PMLA as a carbon source to decompose and digest malate. Among them, a 43-kDa PMLA hydrolase from *Comamonas acidovorans* showed similar enzymatic activity to that of *P. polycephalum*’s, including the similar cleavage of PMLA from one end to the other and the absence of metallo- or serine-esterase activity, whereas its pH optimum was lifted to 8.1 and cellular localization was confined in the outer membrane [52].

## Bioproduction of PMLA and its characterizations

**Fig. 4:**
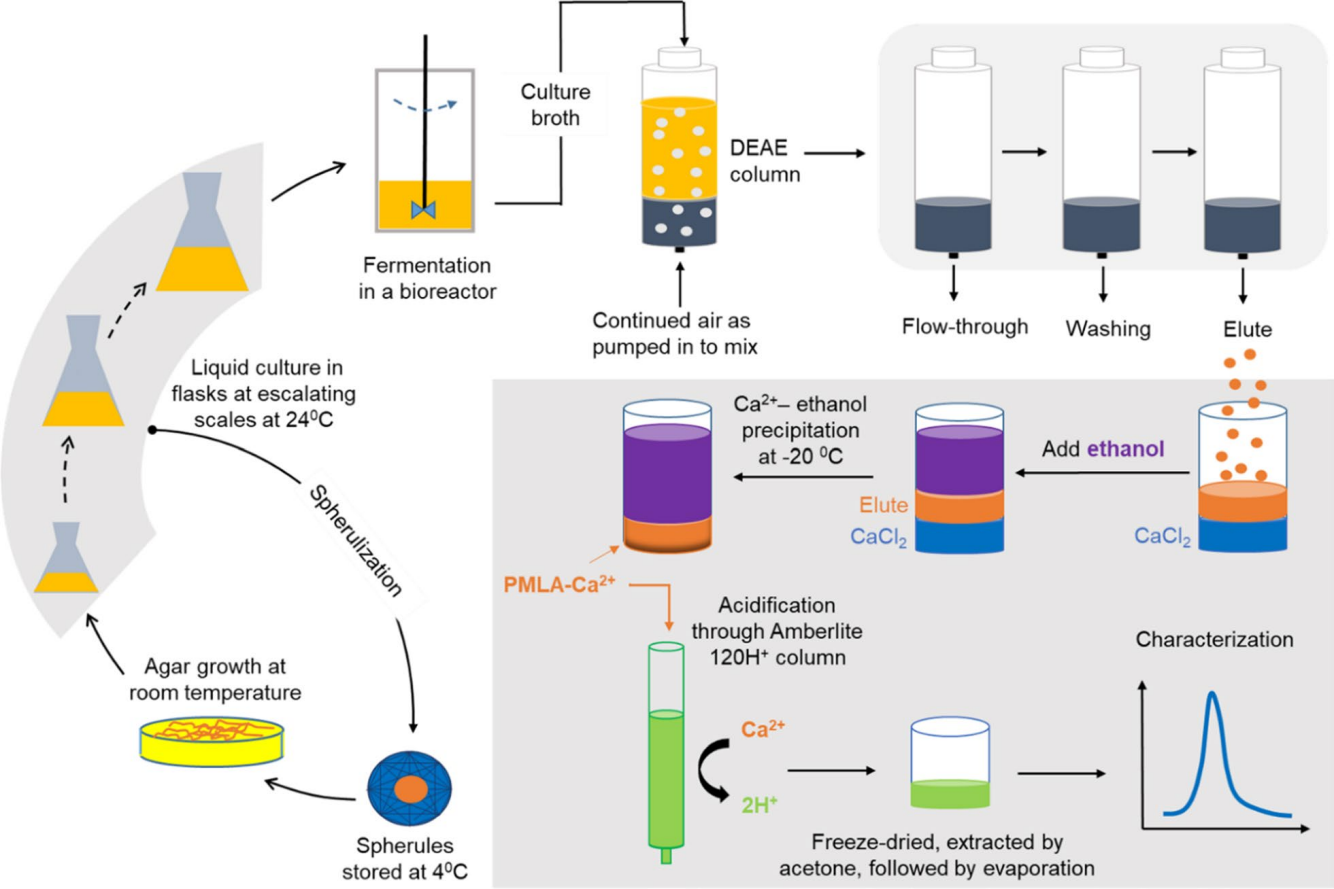
Schematic presentation of PMLA bioproduction by plasmodia of *P. polycephalum*. Spherules, originally kept at 4 °C, are activated to grow on agar before the microplasmodia are removed for liquid culture in 100-, 500-, and 2000-mL-volume flasks with gradually increasing volumes, ending in a typical 10-L fermentation in a bioreactor. During these amplification liquid-culture steps, spherules can be prepared for storage. The resulting culture broth from fermentation is passed through DEAE-cellulose columns, followed by washing and elution [59]. PMLA-Ca^2+^ is then acquired by mixing the elutes with a CaCl_2_ solution, precipitated by adding ice-cold ethanol, further collected, lyophilized to be redissolved in water, and finalized by acidification using an Amberlite ion exchange resin to yield PMLA-H^+^. This final PMLA-H^+^ product needs to be well characterized before its use in biological applications

Until PMLA synthetase can be fully isolated and well characterized, the production of recombinant PMLA in industrial microbes through metabolic or fermentative engineering is unattainable. To date, PMLAs have been produced and extracted from a variety of microorganisms; for example, PMLA bioproduction by the plasmodia of *P. polycephalum* begins with the activation of a spherule, a quiescent plasmodium that can be stored at 4 °C for years, followed by growth on solid agar and then in liquid culture to increase its concentration before its amplification via bioproduction in reactors (Fig. 4) [59]. The culture broth that contains the biosynthesized PMLA is passed through the ion-exchange column for retention of PMLA and removal of many biomolecular impurities, including proteins, nucleotides, and other carbohydrates, followed by repetitive washing and elution of PMLAs [59, 60]. Usually in its salt form (e.g., Ca^2+^ salt), PMLA is finally obtained through a series of purification steps, typically by ethanol precipitation, ion-exchange acidification, acetone extraction, etc. [48]. In addition, fermentation using different strains of *A. pullulans* to produce PMLA follows a similar procedure, except for that the purification may require extra steps to remove pullulan or/and heavy oils as the major coproducts [61]. PMLA bioproduction in fungi is strain-specific, normally showing higher yields and lower *M*_*w*_ when compared to its bioproduction from *P. polycephalum*.

A diversity of saccharides (e.g., glucose, sucrose, fructose, and xylose) can be utilized by *A. pullulans* as a carbon source for metabolism in the cytoplasm and mitochondrial matrix, producing malate for PMLA synthesis; in contrast, *P. polycephalum* can only utilize d-glucose [62–65]. Genomic sequences revealed that *A. pullulans* varieties encode a large number of enzymes responsible for various sugar degradation or transportation, including polysaccharide lyase, glycosyltransferase, glycoside hydrolase, carbohydrate esterase, and carbohydrate-binding modules [66]. Fermentation using *A. pullulans* can therefore sustain an economical PMLA production by sourcing renewable plant-based materials and recyclable environmental wastes [46]. Regardless of the variety of utilizable sugars, d-glucose is the most efficient carbon source for PMLA bioproduction. As research has shown that nitrogen limitation promotes PMLA biosynthesis, comparative transcriptomics and proteomics in *A. pullulans* in environments with restrictive and excess exogenous nitrogen concentrations have revealed an array of genes that could upregulate the synthesis of PMLA under nitrogen stress, particularly those in the target of rapamycin pathway, such as *Gat1* [67, 68]; therefore, gene targeting and nitrogen regulation may uncouple PMLA biosynthesis and cell growth, as both compete for glucose as a carbon source. Similarly, by lowering nitrogen uptake while keeping cell growth to a minimum, PMLA productivity (the amount of PMLA produced per unit of cell biomass) increased in the nongrowing microplasmodia of *P. polycephalum *[32]. Moreover, PMLA bioproduction and cell growth were decoupled in *Aureobasidium sp.* when CuSO_4_ was added into the preparative cell culture (but not into the reaction mixture), possibly because CuSO_4_ initially triggered some enzymatic activity involved in the PMLA biosynthetic pathway, although its presence eventually decelerated cell growth [69].

As shown in Fig. 3, the addition of CaCO_3_ into basal medium serves as a switch from the intramitochondrial oxidative pathway of PMLA biosynthesis to the cytosolic reductive pathway, enhancing the fixation of CO_2_ by pyruvate carboxylase to continue malate production without involvement of the TCA cycle. Theoretically, for each molecule of glucose consumed, more malic acid could be generated in the reductive pathway than in the oxidative pathway; thus, carbonate also serves as a supporting carbon source of significant importance. In *P. polycephalum*, the optimal productivity of PMLA was achieved in the presence of CaCO_3_ when biomass was slightly increased and in this case the addition of intermediates of the TCA cycle (e.g., fumarate, succinate) did not contribute to enhanced productivity [70]. The buffering effect of carbonate not only promotes the availability of dissolved CO_2_ but also adjusts the acidity of the culture medium to prevent a sudden drop in pH that could undermine PMLA production. In *A. pullulans*, a variety of strains has been reported to produce PMLA with high yields in the presence of CaCO_3_, whereas the omission of CaCO_3_ in cell culture leads to an extracellular accumulation of other products, such as pullulan and lipids, rather than PMLA [11, 71, 72]. Intriguingly, replacement of CaCO_3_ with K_2_CO_3_ or Na_2_CO_3_ may either suppress the production of PMLA or shorten the polymeric length, accenting the additional functions of Ca^2+^ in two ways: (1) via the prompt precipitation of PMLA in the culture by the formation of Ca^2+^-PMLA, thereby evading hydrolase degradation; and (2) by possibly involving Ca^2+^ signaling in support of PMLA synthetase activity [47, 61, 73].

The optimized inoculation for PMLA bioproduction requires temperatures of 20–25 °C plus pH = 4.0 for *A. pullulans* strains [74] or temperatures of 24–28 °C plus pH = 5.5 for *P. polycephalum *[70]. Sufficient airing and appropriate stirring are crucial for the reactor settings [75]. The addition of a nonionic surfactant (e.g., Tween 80) into *A. pullulans* cultures at the cell growth stage can increase PMLA productivity by partially solubilizing the plasma membrane and upregulating a variety of metabolic enzymes involved in PMLA biosynthesis and cellular energetics [76]. PMLA producers from various marine and terrestrial habitats have been found and isolated for high-yielding strains [72, 77]. Simultaneously, enzymes governing in vivo malate production have been overexpressed by genetic modulation or exogenous stimulation to promote PMLA productivity, such as pyruvate carboxylase and malate synthase [78, 79]. In addition to optimizing the fermentation process, researchers have implemented new techniques, such as solid-state fermentation and membrane-assisted devices, to improve PMLA productivity and lower the production cost [80, 81].

For quality control, bioproduced PMLA can be quantitatively assessed by a nonspecific colorimetric method when the ester bonds of PMLA react with hydroxylamine/Fe(III) to form Fe(III)hydroxamate, giving a strong absorbance at 540 nm; the quantitative assessment can also be done using a specific photometric/fluorometric measurement of simultaneous NADH (absorbance at 340 nm or fluorescence emission at 455 nm upon excitation at 340 nm) when malate dehydrogenase fully converts the resulting l-malate from the complete hydrolysis of PMLA [82]. Bioproduced PMLAs with comparatively high *M*_*w*_ from *P. polycephalum* or low *M*_*w*_ from *A. pullulans* demonstrate β-type linearity in the polymeric chain. *M*_*w*_ with polydispersity can be relatively determined by viscometry, gel permeation chromatography, or high-pressure liquid chromatography, when choosing the calibration polymers is critical. It also can be absolutely determined by osmometry, light scattering, mass spectrometry, etc., if no presumption of polymer structure and elasticity is made. PMLA complexes can also be further quantitatively analyzed after backbone cleavage by aqueous ammonia and individual assessment of each component [83].

## PMLA as a promising nanomedicine for disease diagnosis and therapy

Since its discovery, PMLA has been identified as a carrier that shuttles between the nucleus and the cytoplasm to relocate nuclear proteins [5, 43]. PMLA in bacteria or animals has not been identified, but PMLA can be used by all living organisms as a nutrient source to produce malic acid. As the only naturally occurring water-soluble polyester, PMLA has an abundance of carboxylate groups as pendants along its polymeric backbone (over 8 mmol/g PMLA), making it stable in aqueous solutions but reactive for the attachment of functional moieties.

### Suitability of PMLAs as drug carriers

The toxicity and immunogenicity of chemically synthetic PMLA (*M*_*w*_ = 17–25 kDa) in experimental animals were tested with no acute nor undesired effects, whereas its pharmacokinetics showed a short half-life (within minutes), and its biodistribution demonstrated a major accumulation in the kidneys and a rapid excretion in the urine (70% in 1 h and 90% in 3–6 h) with a low but persistent liver uptake (beyond 24 h) post intravenous (i.v.) injection [84, 85]. This is consistent with the later finding that PMLA of *M*_*w*_ = 100 kDa from *P. polycephalum* stayed fully ionized in aqueous solutions and retained an open-coil conformation with a hydrodynamic diameter of 6.6 nm [86], the cut-off size for kidney glomerular filtration and urinary excretion [87]. These physiochemical features make PMLA an unrivaled delivery vehicle, especially in human urinary system. For instance, PLMA-derived nanoparticles can be filtrated through glomerular and enter the urinary tract, finally excreted through the urethra by urine. During this urination process, PLMA-derived nanoparticles could exert their medicinal role in the diagnosis and treatment of urinary malignancy (e.g., urothelial carcinoma) or other diseases, such as urinary tract infections. Alternatively, PLMA-based nanoparticles could be instilled retrogradely into the urinary bladder through the urethra to interact with the urothelium directly, in a similar way where docetaxel-loaded polyglycerol-complexed nanoparticles were shown to inhibit tumor growth in an orthotopic murine model of bladder cancer by increasing intratumoral drug accumulation [88].

Synthetic PMLA was implemented as a drug carrier for the first time when fluorescent dyes or anti-neoplasm drugs were covalently linked to its pendant carboxylic acids via carbodiimide-induced amide bond formation [8, 89]. The attachment of small-molecule drugs/imaging agents to this nanosized carrier alters the cell uptake route from free drug diffusion to endocytosis of the polymer–drug conjugates, thereby increasing the intracellular or even intranuclear (e.g., for certain DNA alkylation reagents) drug concentration; however, this therapeutic improvement was downplayed by the premature release of the drug, owing to the fast hydrolytic degradation of the PMLA backbone. Regardless of its natural or synthetic origin, PMLA shows a similar hydrolytic mechanism at physiological condition (i.e., pH = 7.4, 37 °C, bodily ionic strength) in which it occurs the random rupture of main-chain ester bonds and concurrent formation of various oligomer intermediates [90, 91]. The non-enzymatic hydrolysis obeys the first-order kinetics, accelerated by increased temperature, acidified pH, and prolonged polymer size [90, 92]. Concomitantly, biosynthetic PMLA of *M*_*w*_ = 9 kDa and 2% (w/v) in water has pH = 2.0, being a strong polybasic acid with p*K*_*a*_ = 3.6 [93]. Complete hydrophilicity and negative charge at the physiological pH make PMLA in polyanion form and thus unsuitable for cellular uptake as it trespasses through cell membranes of negative surface charge and lipid bilayer compositions with extremely low efficiency.

Nevertheless, the pharmaceutical application of PMLA reveals a new avenue in translational medicine using sustainable polymers from natural sources with no adverse reactions, and research efforts are continuously being made to identify a more suitable PMLA-based delivery platform. To enhance the interaction between the biopolymer and the plasma membrane, the lipophilicity of PMLA-based nanomaterials is intentionally increased by adding different ratios of diazomethane to PMLA for reactions to achieve a partial methylation, resulting in various degrees of esterification and the generation of a nonrandom copolymer PMLA-Me_x_H_100-x_ (where *x* is the percentage of methyl units) [94, 95]. As *x* increased, hydrophobicity increased in the order as follows: PMLA < PMLA-Me_25_H_75_ < PMLA-Me_50_H_50_ < PMLA-Me_75_H_25_ < PMLA-Me, where PMLA-Me_75_H_25_ and PMLA-Me were completely insoluble in water. The same order came forth with the prolonged hydrolysis in saline and plasma, intensified rupture in liposome membrane and augmented cytotoxicity (although this was possibly due to the intracellular degradation of polymers into methanol rather than the polymer per se) [94]. Similarly, the more hydrophobic substitution (different characters or numbers) was introduced to the carboxyl pendant in the PMLA, the slower hydrolytic degradation in the main chain occurred [96, 97]. The explanation might be due to more difficulty in water penetration to more hydrophobic core of polymer particles, or accordingly less mobility in the polymeric chain as a result of more hydrophobic replacement in the pendant carboxylate, evidenced by the higher glass transition temperature (*T*_g_) of fully benzylated PMLA compared with that of fully hexyl PMLA or partially benzylated PMLA [96, 97]. For the same reason, the hydrolytic mechanism for PMLA with hydrophobic substitutions in its side chain follows a “backbone-first and pendant-second” order.

Hydrophobic amino acids or peptides were also conjugated to carboxylic acids in the side chain of PMLA to modulate its hydrophilicity and net charge, thus adjusting PMLA’s interactions with cellular and subcellular membranes in a pH-responsive manner [35, 86, 98]. Inspired by the characteristic amino acid sequences in cell membrane–penetrating peptides, various PMLA–amino acid conjugates were synthesized. The conjugates were screened for length, conformation, lipophilicity, and charge and their membranolytic activity were analyzed under different pHs [35, 86, 98]. Compared with PMLA that remains anionic and hydrophilic under most conditions (i.e., pH > p*K*_a_), the copolymers of PMLA and certain lipophilic amino acids/peptides can adjust their protonation or neutralization according to environmental changes in pH, assembling into particles of ~ 100 nm for an optimized hydrophobic interaction with the lipid membrane to make it permeable [99]. For example, trileucine conjugates of PMLA (with 40% pendant substitution, i.e., PMLA-LLL_40_) exhibited a membranolytic activity in a pH-dependent manner [86]. Trileucine owns three lipophilic isobutyl groups in its side chain and one carboxylic acid at the end; therefore, a neutral pH could keep the terminal of PMLA-LLL_40_ negatively charged as it did for PMLA, preventing its nonspecific protein adsorption and cellular uptake, but an acidic pH (e.g., endosomal/lysosomal pH = 4.5–6.5) made this copolymer neutralized or protonated, thereby facilitating its endosome escape and cytosolic liberation [99].

A final hydrophobic–hydrophilic balance in engineered PMLA copolymers can be achieved by considering the various physicochemical factors of potential binding groups, including their polarity, distribution, density, rigidity, and charge. These determinants are crucial to favor membrane crossing and cellular uptake while inclusive cell viability is maintained. Consequently, intracellular trafficking of nanosized biomaterials counts on those physicochemical properties to successfully dodge unwanted binding and optimally reach cellular or subcellular targets [100]. PMLA, as an efficient platform to carry therapeutic or diagnostic reagents, needs to be carefully designed and properly functionalized to ensure an effective endosome escape, where a pH-dependent membrane rupture occurs in response to the changing acidity in the intracellular environment, thereby obviating enzymatic degradation in the subcellular compartment and fleeing to set free the shipping cargos.

In the diagnosis and treatment of severe human diseases such as malignant tumors, chemotherapy and immunotherapy are the predominant treatment methods; however, there are many drawbacks in their clinical practice. One is that small-molecule anticancer agents with poor water solubility circulate with a short lifetime in the body during which their bioavailability is extremely low [101]. Another is that the complexity and heterogeneity of the cancer microenvironment significantly prevent drug accumulation and action at the tumor site [102]. Given these problems, delivery systems that modify pharmacokinetics in favor of improved pharmacodynamics are greatly desired. Thanks to its excellent water solubility, total biodegradability, non-immunogenicity, and minimal toxicity at maximal dosage, PMLA has an unrivaled biological compatibility, serving as a competent platform for the further encapsulation and delivery of diagnostically and/or therapeutically active molecules to the target site. Moreover, the high chemical reactivity of its abundant pedant carboxylic acid groups guarantees PMLA’s exceptional loading capacity as well as its multifaceted functionality, providing a multipurpose carriage in order to maximize synergism and minimize side effects. In particular, for drug delivery to solid tumors, PMLA-based stimuli-responsive platforms at nanoscale are especially useful as they circumvent the unique features of the tumor microenvironment [3]. These PMLA platforms can enhance the permeability and retention of nanoparticles in irregular blood vessels, promote hypoxia- or redox-mediated drug targeting in the extracellular matrix, and exert a pH-triggered drug payload release in acidic tumor cells.

### Stepwise conjugation through pendant carboxylic acids in PMLA

Through step-by-step chemical synthesis, PMLA extracted from *P. polycephalum* can be combined with different functional fragments (such as polyethylene glycol or PEG, antibodies, peptides, nucleotides, fluorescent tracers, or chemotherapeutic drugs) through direct or indirect (through spacer) conjugation to create a set of biopolymers named Polycefin [12, 103]. Among them, different pendant moieties along the PMLA backbone can be assigned with different biological missions; for instance, PEG can reduce nonspecific protein binding and prolong circulation time in the bloodstream, whereas a specific antibody can be designed to target the pairing receptor of target tumors and deliver a high concentration of drug [103]. Antibodies (such as the transferrin receptor monoclonal antibody, TfR mAb) or membrane-disruptive peptides (such as l-valine or trileucine) help penetrate biological barriers to protect nanoconjugates from early degradation and therefore increase the final drug accumulation at the destination; moreover, antisense oligonucleotides (AONs), synthetic DNA oligomers that can form highly sequence-specific hybrids with target RNA, are attached to inhibit the overexpressed laminin-8 in human glioblastoma multiforme (GBM) [104]. Finally, therapeutic synergism can be achieved as well as visually proven by bonding theranostic segments in Polycefin, including inhibitory nucleic acids, small-molecule chemical drugs, and multimodal imaging agents.

**Fig. 5:**
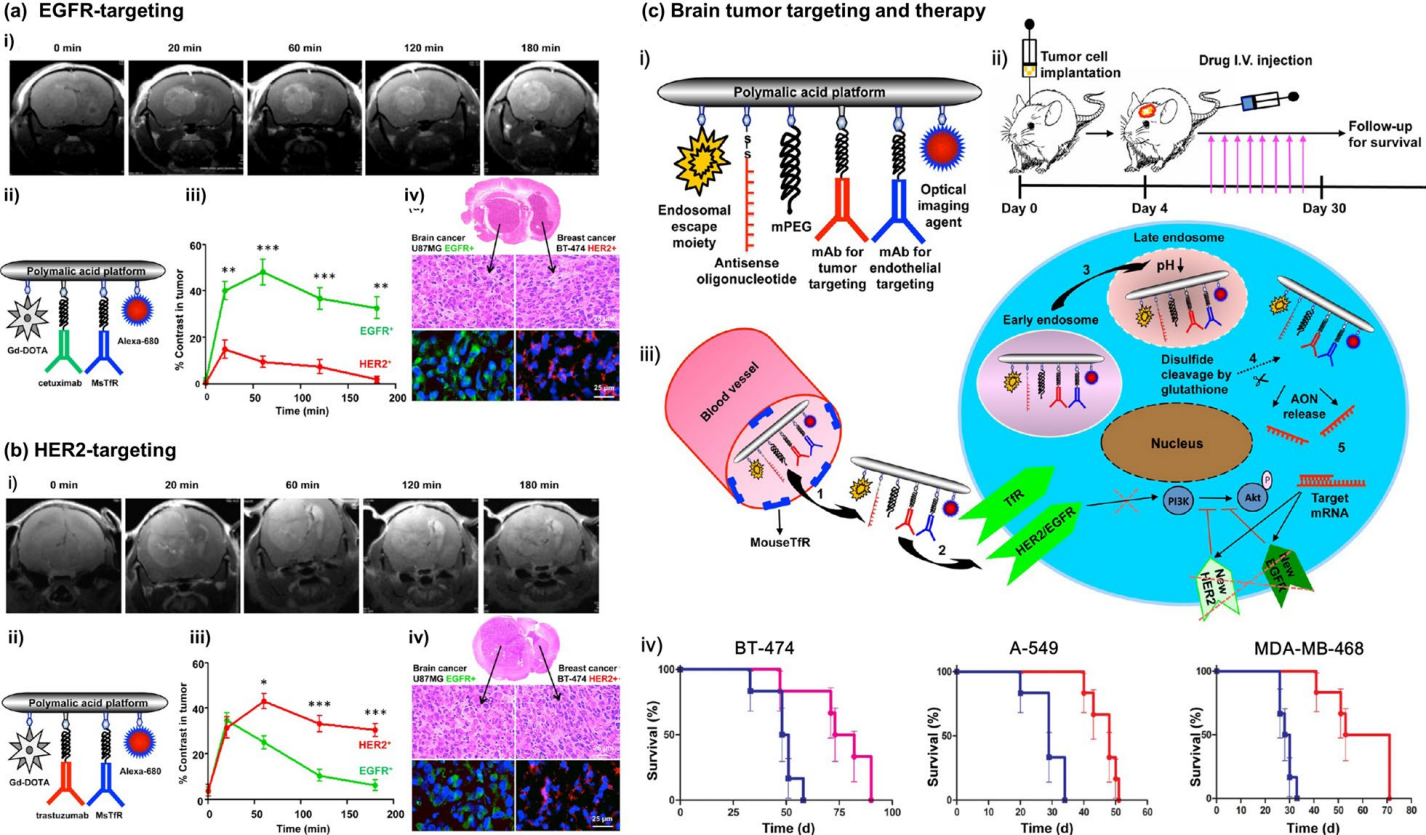
PMLA-based nanoparticles for tumor theranostics. **a** (i) MRI brain scans of mice with double tumors: a GBM (U87MG, EGFR^+^) in the left hemisphere and a metastatic breast cancer (BT-474, HER2^+^) in the right hemisphere after IV injection of (ii) targeted PMLA conjugated with Gd-DOTA/cetuximab/mouse TfR-mAb/Alexa 680, with (iii) quantitative analysis of MRI contrast in tumors and (iv) confirmation of MRI diagnosis by immunohistochemical analysis. Reprinted from ref. [106], with permission from the American Chemical Society, Copyright 2015. **b** (i) MRI scans of mice with double tumors, a primary GBM (U87MG, EGFR^+^) in the left hemisphere and metastatic breast cancer (BT-474, HER2^+^) in the right hemisphere after IV injection of (ii) targeted PMLA conjugated with Gd-DOTA/trastuzumab/mouse TfR-mAb/Alexa 680, with (iii) quantitative analysis of MRI contrast in tumors and (iv) confirmation of MRI diagnosis by immunohistochemical analysis. Reprinted from ref. [106], with permission from the American Chemical Society, Copyright 2015. **c** Schematic presentation of (i) PMLA-based nanodrugs, (ii) stereotactic implantation of brain tumors, and (iii) proposed mechanism of action. (iv) Kaplan–Meier animal survival curve for treatment of HER2^+^ BT-474 brain metastasis, EGFR^+^ A-549 brain metastasis, EGFR^+^ triple-negative MDA-MB-468 brain metastasis. The corresponding PMLA nanoconjugates improved survival by 57%, 66%, and by 114%, respectively (from left to right). Reprinted from ref. [106], with permission from the American Chemical Society, Copyright 2015

Using naturally derived PMLA as a nanoplatform, a series of Polycefin-based imaging and treatment substances was designed and fabricated with the goal of penetrating the blood–brain barrier (BBB) or blood–brain tumor barrier (BBTB) for diagnosis and therapy of neurological disorders, including primary and metastatic brain tumors and Alzheimer’s disease. Purified PMLA from the culture medium of *P. polycephalum* of *M*_*w*_ = 80 kDa with a polydispersity of 1.3 was covalently linked to gadolinium-DOTA (Gd-DOTA, a contrast enhancement tracer based on Gd) and curcumin [105]. These complexes, having a sub-10-nm size (8.6 nm), enabled the magnetic resonance imaging (MRI) detection of amyloidal beta (Aβ) plaques in ex vivo brain samples of human and mouse models of Alzheimer's disease [105]. Later, dual-imaging modes of the fluorescent dye Alexa 680 and the MRI reagent Gd-DOTA were added to a PMLA scaffold, and the complex was further conjugated with therapeutic monoclonal antibodies (trastuzumab for HER2 targeting and/or cetuximab for EGFR targeting) in order to easily differentiate a simultaneous diagnosis of HER2^+^ and EGFR^+^ brain tumors (Fig. 5) [106].

**Fig. 6:**
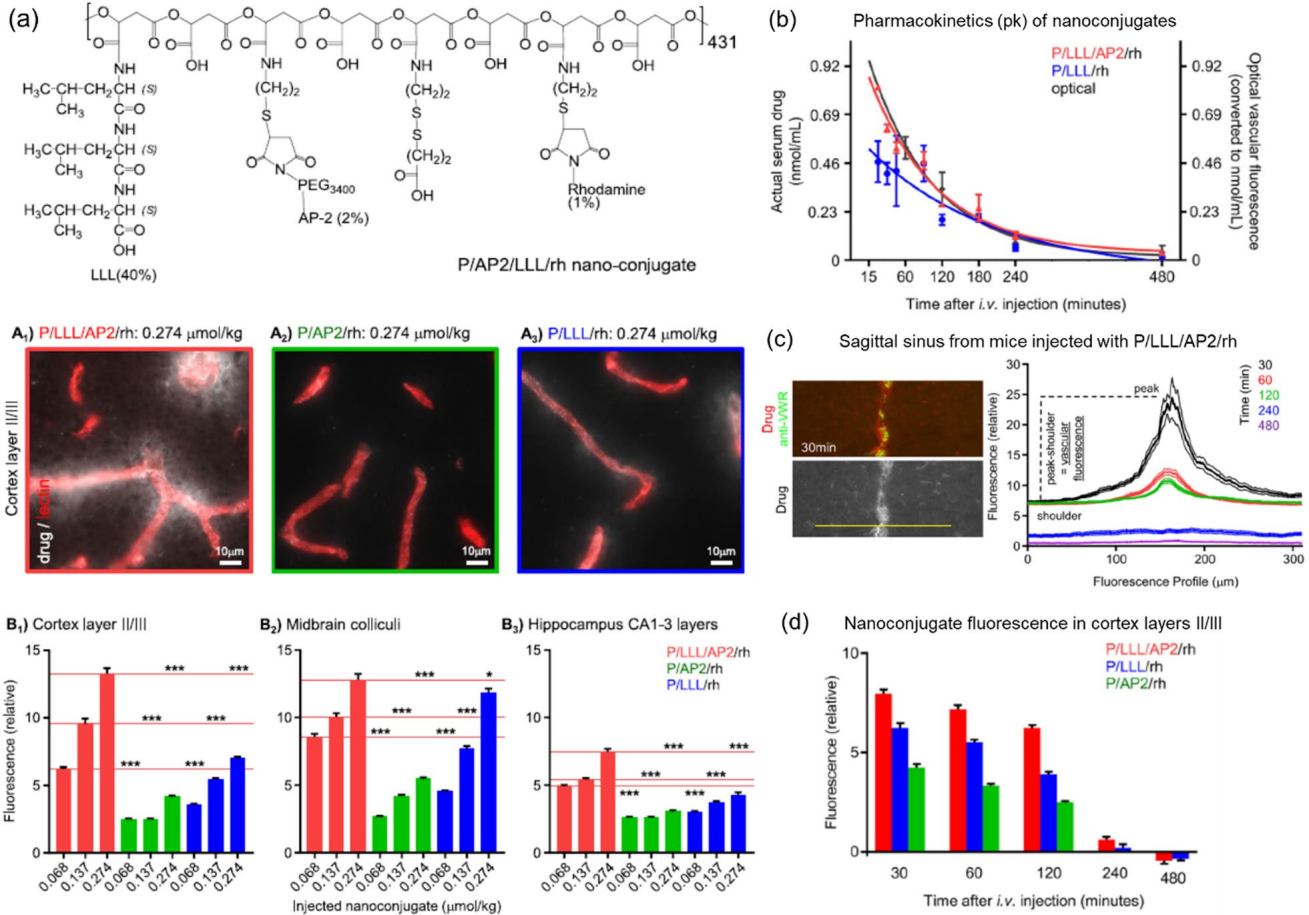
P/LLL/AP2/rh nanoconjugates for BBB penetration. **a** Chemical composition of P/LLL/AP2/rh, optical imaging data showing nanoconjugate permeation of the cerebral cortex (A_1-3_) and average nanoconjugate fluorescence in brain parenchyma (B_1-3_). **b** Pharmacokinetics of nanoconjugate in serum (P/LLL/AP2/rh and P/LLL/rh) and brain tissue (optical imaging of the sagittal sinus blood vessel). **c** Vascular fluorescence intensity profile (right) for the sagittal sinus vessel as indicated with a yellow line (only 30 min drug accumulation shown in the left). **d** Time dependence of nanoconjugate fluorescence intensity in brain parenchyma. Reprinted from ref. [107], with permission from the American Chemical Society, Copyright 2019

**Fig. 7:**
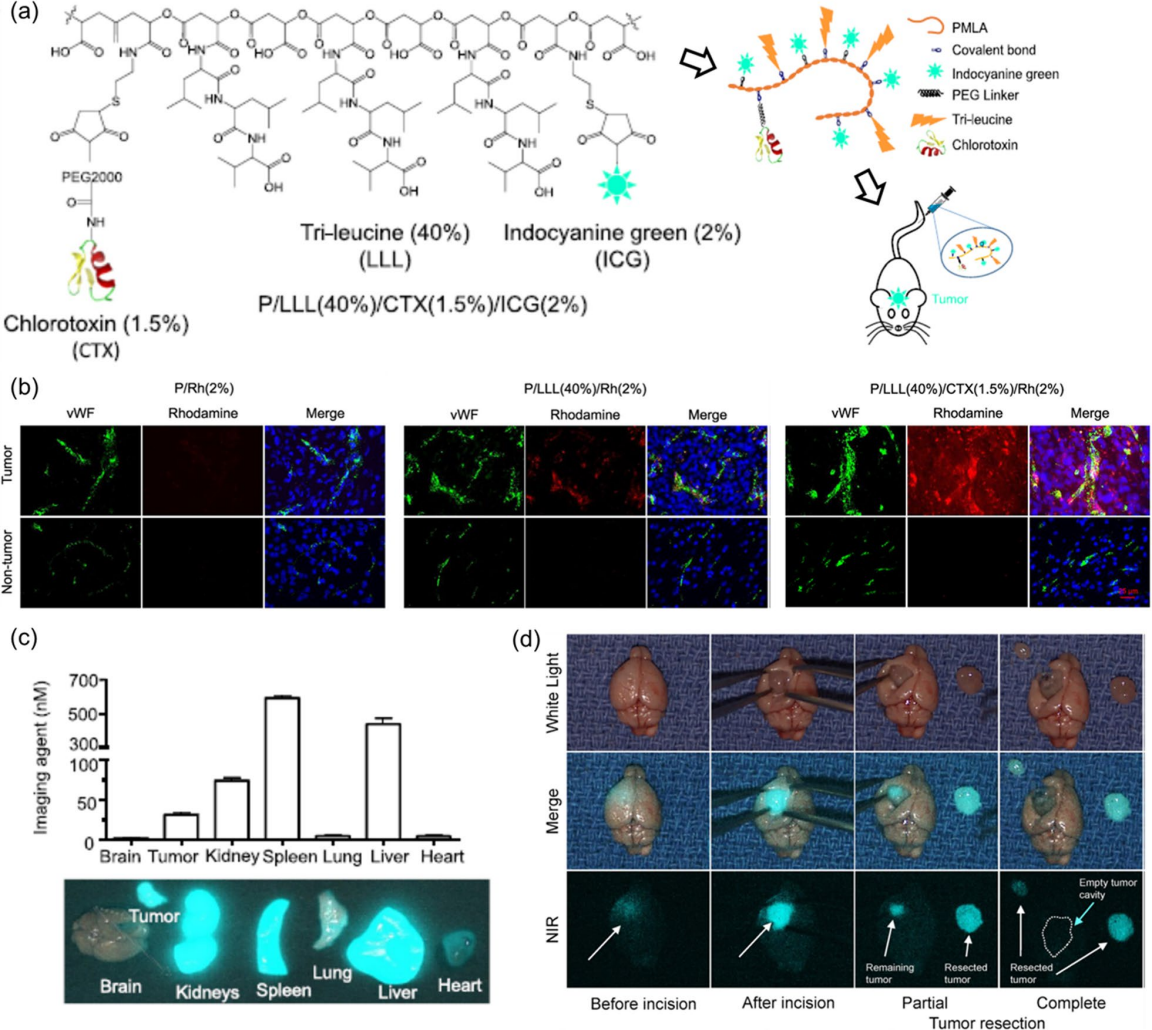
P/LLL/CTX/ICG nanoconjugate for NIR-guided resection of brain tumor. **a** Composition of P/LLL/CTX/ICG and its injection into murine model of brain tumor. **b** Ex vivo fluorescence microscopy showing extravasation of P/LLL/CTX/ICG across BBB and intense distribution outside blood capillary and next to nuclei in tumor cells. **c** Biodistribution of P/LLL/CTX/ICG and **d** Real time NIR-imaging and surgical resection of GBM, 4 h after IV injection into mouse tail vein. Reprinted from ref. [110], with permission from Elsevier, Copyright 2019

Recently, by switching the navigator antibody in Polycefin, a BBB-penetrating peptide Angiopep-2 (AP-2) was chemically attached onto the PMLA base (*M*_w_ = 50 kDa), together with LLL to facilitate an endosome escape and rhodamine (rh) for fluorescent tracing [107]. Having a hydrodynamic diameter of < 5 nm, this new version of Polycefin (PMLA/LLL/Angiopep-2/rhodamine, i.e., P/LLL/AP2/rh), unlike either P/LLL/rh or P/AP2/rh that could not substantially penetrate BBB, showed a rapid accumulation in brain regions 30 min after i.v. administration and a fast clearance after 4 h (Fig. 6), demonstrating a promising neurological delivery platform [107]. Moreover, AP-2–guided PMLA conjugates with multiarm Gd contrast agents, which measured 15.8–20.5 nm in size, also showed a successful delivery to mouse glioma models and an enhanced brain tumor detection [108]. TfR- or Ap-2–directed PMLA in covalent conjugation with checkpoint inhibitors, including cytotoxic T-lymphocyte–associated antigen 4 (CTLA-4) and programmed cell death 1 (PD-1) antibodies, was successfully delivered into brain tumor cells, triggering local immune responses and prolonging the survival of intracranial GBM-bearing mice [109]; similarly, chlorotoxin (CTX), which showed exceptional BBB-penetrating and specific GBM-binding ability, was conjugated to Polycefin together with a clinically approved near-infrared (NIR) fluorescent tracer, indocyanine green (ICG), making P/LLL/CTX/ICG nanoconjugates, and was injected i.v. into GBM-bearing mice for an NIR fluorescence-facilitated brain tumor resection, showing a high precision and the completion of tumor removal (Fig. 7) [110].

**Fig. 8:**
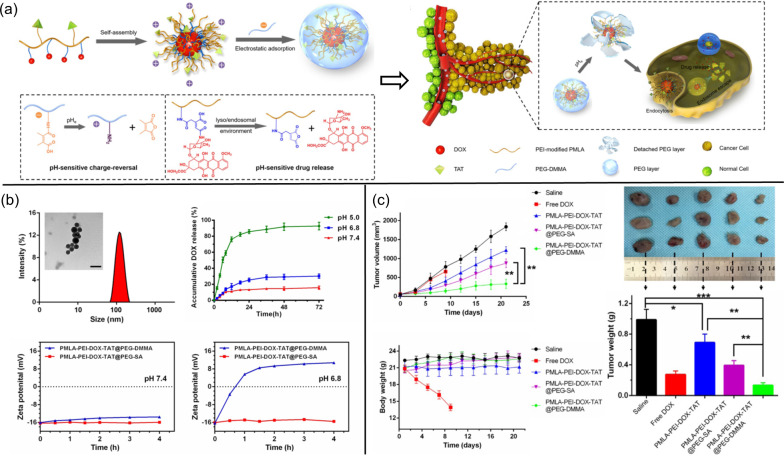
PMLA-PEI-DOX-TAT@PEG-DMMA nanocomplex for anticancer activity. **a** Illustration of PMLA-PEI-DOX-TAT@PEG-DMMA prepared for effective drug release in cells. **b** Characterization of PMLA-PEI-DOX-TAT@PEG-DMMA. **c** The antitumor effect of nanocomplex on nude mice bearing A549 cells subcutaneously. Reprinted from ref. [113], with permission from Ivyspring International Publisher, Copyright 2017

Similar to the design of Polycefin, many other PMLA-derived nanoconjugates have been developed for cancer targeting and treatment, most of which are based on the use of chemically synthetic polymers [111, 112]; for instance, one PMLA nanocomplex was constructed via stepwise chemistry: first, an amount of proton-sponge material polyethylenimine (PEI) was used to reverse the negative charge of PMLA, thus becoming positive; next, doxorubicin (DOX) and the transactivator of transcription (TAT) peptide were conjugated to the pendant carboxylic groups in PMLA through a pH-responsive linker and a primary amine-contained maleimide spacer, respectively, which formed PMLA-PEI-DOX-TAT micelles in aqueous solution; finally, six-armed PEG was conjugated to 2,3-dimethylmaleic anhydride (DMMA) or succinic anhydride (SA) and then added to solutions of PMLA-PEI-DOX-TAT micelles to dock on their outer surface through electrostatic adsorption, yielding PMLA-PEI-DOX-TAT@PEG-DMMA and PMLA-PEI-DOX-TAT@PEG-SA nanocomplexes (Fig. 8) [113]. PMLA-PEI-DOX-TAT@PEG-SA was applied as a non-charge-reversal control because of its structural similarity (but reverse pH-sensitivity) to PMLA-PEI-DOX-TAT@PEG-DMMA (Fig. 8b) [113]. After injection into the bloodstream, the shielding effect of PEG and the negative charge of the nanocomplexes at a physiological pH prevented 120-nm-sized particles from immediate recognition and clearance by the reticuloendothelial system, thereby improving their pharmacokinetics and allowing access into the acidic tumor microenvironment; DMMA (but not SA) was responsively disposed of, thus providing the positively-charged TAT-guided nanoparticles an advantage in penetrating tumor cells and unloading DOX drugs for tumor inhibition (Fig. 8c) [113].

It is evident, therefore, that PMLA can serve as a flexible and versatile platform for further add-ons; nevertheless, these additions require a multi-step synthesis of different functional groups and the repetitive purification of intermediate products, resulting in a small yield of a final product potentially contaminated with unwanted side products. At the same time, the aqueous medium needs to be optimized for PMLA conjugation to prevent hydrolysis of the PMLA backbone, which would partially or completely negate functionalization. This optimization is especially challenging in the manufacturing industry and for biosafety assessments before clinical trials. Furthermore, through conjugation to form covalent bonds with pendant carboxylic acids, the chemical structures of the various attaching moieties might not remain intact, causing a possible loss of function to an unknown degree.

### Simultaneous modification on pendant carboxylic acids in PMLA

Different from the stepwise conjugation described above, a simultaneous modification through chemical transformation or noncovalent binding of pendant carboxylic acid groups can be used to form PMLA-based nanostructures. With this method, after PMLAs are purified from fermented microorganisms, their malic acid units are transformed into partial blocks of opposite hydrophilicity (e.g., via benzylation) or are bound to electrolytes of opposite charge (via cationic complexation). The PMLA-based nanostructures obtained are thus restabilized as nanoscale platforms, enabling the loading and controlled release of pharmaceutical molecules.

Methylated PMLAs were obtained in a typical one-pot synthesis in which different ratios of diazomethane over PMLA (*M*_*w*_ = 34 kDa) in acetone were mixed for the reactions, and partial esterification of the pendant carboxylates was accomplished at random [94]. In aqueous solutions, the size of the newly formed polymer nanoparticle was determined to be between 3.0 and 5.2 nm; comparatively, from PMLA to a more methylated PMLA, the surface charge became less negative and the rate of hydrolytic degradation beginning with the breakage of the methylated carboxylate groups followed by hydrolysis of the PMLA backbone was reduced [94]. Hydrophobic drugs can be encapsulated within the methylated PMLA region through hydrophobic interactions, and their release occurs with the hydrolysis of the ester bonds, a process that is independent of cargo load but modifiable according to the hydrophobicity of copolymer [95]. This drug release can be further controlled or accelerated by the addition of certain external stimuli to the biological milieu, including changes in pH, ionic strength, redox, etc.

**Fig. 9:**
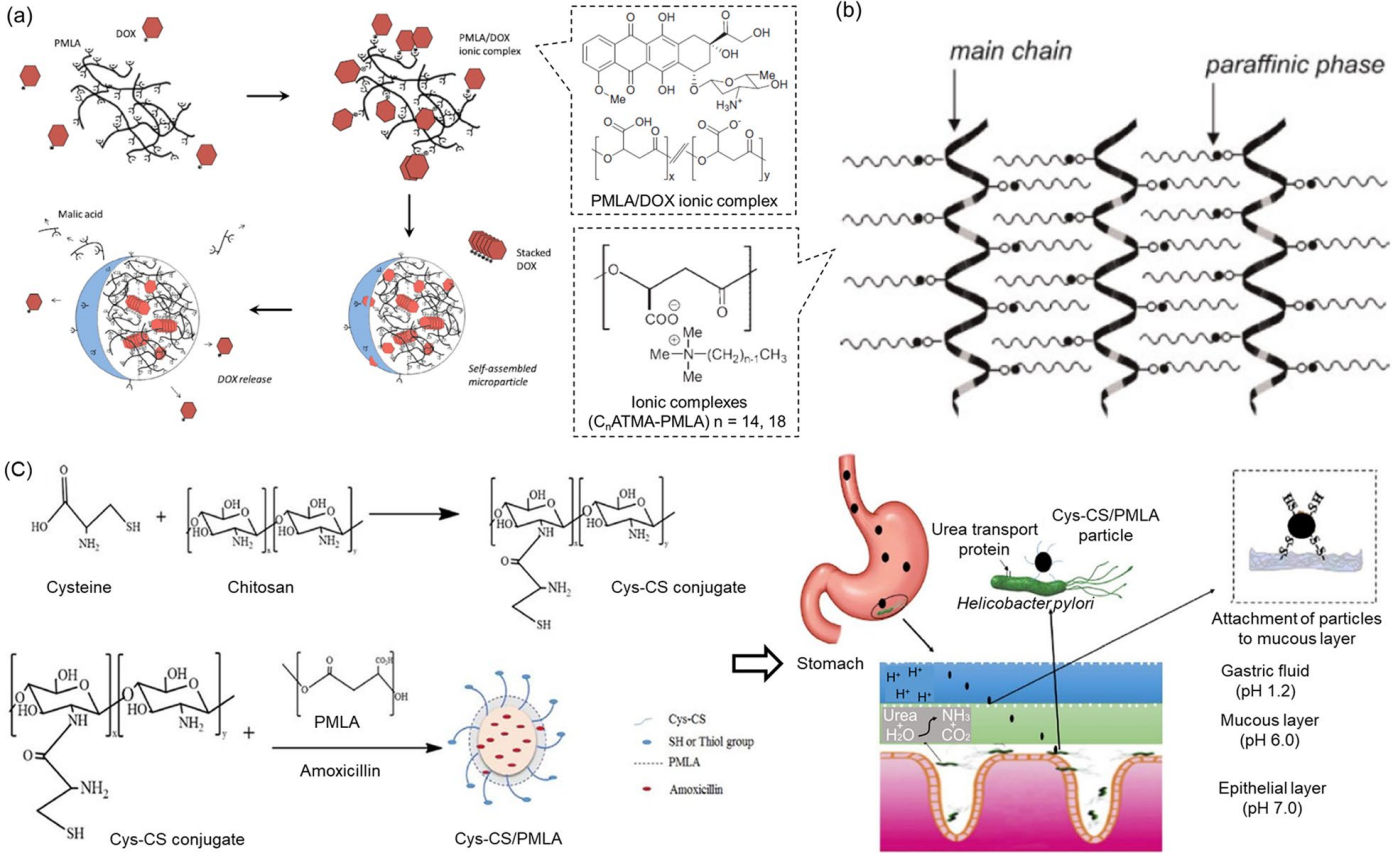
PMLA-based ionic complexation. **a** Schematic illustration of PMLA/DOX particle formation and drug loading and release. Reprinted from ref. [114], with permission from Elsevier, Copyright 2014. **b** Supramolecular structure of C_n_ATMA-PMLA ionic complex. Reprinted from ref. [115], with permission from Wiley–VCH, Copyright 2007. **c** Formation of Cys-CS/PMLA particles and their pH-sensitive release of antibiotics to treat *Helicobacter pylori* in the stomach. Reprinted from ref. [117], with permission from Elsevier, Copyright 2018

Polymalic acid under most biological conditions becomes a polyanion that possesses a number of carboxylate ions, which can easily interact with cationic moieties to form stable complexes [114]. In a typical case at pH 4.0, different moles of positively-charged DOX (p*K*_a_ = 8.6) were added into aqueous solutions of PMLA with a fixed mole (in units of malic acid; i.e., DOX/PMLA = 0.1:1, 0.25:1, and 0.5:1). The increasing amount of DOX made the solution change from colloidal suspension to reddish precipitation, and > 90% added DOX formed ionic complexes with carboxylic acids of PMLA via their amines [114]. After DOX was added into the PMLA solution, this hydrophobic drug stayed in the inner core of the formed spheres, whereas the hydrophilic polymer constituted the outer surface through self-assembly (Fig. 9a). With more DOX partaking in the interaction, the attached drug itself formed π–π stacking (a strong cohesive force that significantly shrinks the inner core to make nanospheres) between their aromatic sheets [114]. The loading efficiency of DOX was shown to be almost three times higher than that using the physical encapsulation of DOX by methylated PMLA; in comparison, the drug released by the spherical PMLA/DOX ionic complexes was governed by both changes in pH and ionic strength, through a mechanism in which an acidic pH could quickly degrade PMLA’s outer surface in the spheres and, to a lesser extent, the increased concentration of electrolytes would diminish the ionic bonding between DOX and PMLA [114].

Alternatively, anionic PMLA can first react with cationic compounds to form a supramolecular assembly where its property of loading and releasing drugs becomes further adjustable by choosing a variety of cationic counterparts. In a pilot study, alkyltrimethylammonium (ATMA) surfactants with different lengths of alkyl chains (i.e., C_n_ATMA, where *n* represents the number of carbon atoms in the alkyl chain) were chosen to form ionic complexes with PMLA (C_n_ATMA-PMLA). The ionic complexes were studied to determine their interplay with antibiotics for drug loading and release [115]. Results showed that the ionized surfactants in the aqueous solutions were adsorbed by polyacids through electrostatic interaction, and, between the PMLA backbones, they were intercalated by hydrophobic surfactants with long alkyl chains, showing an ordered nanostructure with a paraffinic phase (Fig. 9b) [115]. For this reason, a higher degree of hydrophobicity, which is achieved with a longer alkyl chain in C_n_ATMA, helps to prevent a water/enzyme attack that would break down the ester bonds in PMLA backbone, resulting in a slower hydrolytic or enzymatic degradation of C_n_ATMA-PMLA and consequently releasing single units of erythromycin-bound malates at a slower rate (i.e., the degradation and erythromycin-releasing rate both followed the order PMLA > C_14_ATMA-PMLA > C_18_ATMA-PMLA) [115].

Polymalic acid of a high *M*_w_ (205.4 kDa) produced from the liquid fermentation of *Aureobasidium pullulans var. pullulans* MCW, was mixed with cysteine-conjugated chitosan (Cys-CS) via ionic gelation at different weight ratios to form particles of different sizes and surface charges [116]. Because of electrostatic association, positively charged Cys-CS/PMLA particles formed intact spheres at pH = 1.2 (simulating gastric acid), dramatically swelled at pH = 6.0 (simulating gastric mucosa), and completely dissociated at pH = 7.0 (simulating *Helicobacter pylori*). These particles were chosen to load antibacterial amoxicillin, further showing a pH-triggered release to inhibit the growth of *Helicobacter pylori* while leaving stomach cells unharmed (Fig. 9c) [117]. A strong intermolecular binding between thiolated Cys-CS/PMLA and glycosylated mucin was also confirmed, displaying an excellent mucoadhesive potential for enhanced delivery [116]. These Cys-CS/PMLA particles provide the scaffold for orally administered drugs designed to treat stomach bacterial infections. The subsequent inclusion of biosurfactants (rhamnolipids) or carbon dots into the CS/PMLA system showed a superior loading capacity over 70% with a particle size of ~ 200 nm and an antimicrobial effect against *H. pylori* of 99% [118, 119].

In addition, a biodegradable thin film was prepared after a layer-by-layer assembly of PMLA and chitosan, revealing an excellent capacity for the controlled release of proteins, including lysozyme and basic fibroblast growth factor (bFGF) [120]. To incorporate proteins, a tetralayer architecture (of chitosan/PMLA/protein/PMLA)_n_ was assembled, having a thickness of 69.2 nm and 10.7 nm for lysozyme and bFGF films, respectively (The bFGF film per tetralayer was significantly thinner than the lysozyme film because of the more potent nature of bFGF; therefore much less protein was able to be applied to the film), and their film growth exhibited a high degree of linearity between film thickness and the number of tetralayers. This tetralayer architecture demonstrated an exceptional diffusiveness between the layers of film, which would be advantageous for cargo release [120]. It was shown that the chitosan addition to the (chitosan/PMLA/protein/PMLA)_n_ tetralayer prevented the abrupt release of biologics in the case of (protein/PMLA)_n_ bilayers, sustaining a timed release due to the hydrolytic degradation of PMLA and the accompanied dissociation of ionic complexes in response to changes in environmental pH and ionic strength; comparatively, dissembled chitosan could preserve the activity of the released proteins by preventing them from being denatured or proteolyzed [120].

In PMLA-based ionic complexation, whether PMLA forms stable complexes directly with positively-charged functional compounds of low *M*_w_ (e.g., small-molecule drugs) or first interacts with oppositely charged polyelectrolytes of high *M*_w_ and then with functional compounds, the complexes undergo a completion of reactions in aqueous solutions. These series of reactions aid in unleashing more absorbed therapeutic or diagnostic reagents with the addition of stimuli or by stimuli-triggered self-degradation of PMLA main chain compared with free drug diffusion. Accordingly, PMLA hydrolysis, together with the dissociation of the ionic complex, liberates the drug-bound malate units.

## Conclusion and perspective

Here we review the recent advances in the chemical synthesis and bioproduction of PMLAs, their physicochemical and physiochemical characteristics, their biomedical applications as delivery platforms for disease diagnosis and treatment, and the unique chemical methods used to engineer PMLA-based nanomaterials. Produced from biological sources rather than chemical reactions, PMLA has several advantages as a unique platform for theranostic delivery in the clinical translation. Firstly, manufactured through fermentation engineering of microorganisms, PMLA is a biorenewable natural product with promising full-scale industrialization. Secondly, water solubility of PMLA is unrivaled when compared with water-insoluble biopolymers, such as cellulose, polyhydroxybutyrate, etc., or biopolymers that are only water soluble in salt form, such as polyglutamic acid. More importantly, this excellent water solubility is inherited by many PMLA-derived complexes, maintaining their suitability as delivery platforms. Thirdly, pendant carboxylic acids along the main chain of PMLA offer several opportunities for further covalent conjugation or ionic complexation, making PMLA a resourceful carrier for therapeutic and diagnostic delivery. Fourthly, the release mechanism of a PMLA carrier is mostly due to the hydrolytic breakage of the ester bonds in its backbone, although it could be complemented with the dissociation of other intermolecular forces, such as ionic bonds. The timing of this release profile greatly avoids a premature cargo leakage before delivery to the diseased lesion. The degradative or hydrolytic rate of PMLA under physiological conditions could be favored by pharmacokinetics. Lastly, the full biodegradability can make PMLA adaptable to many formulations, including orally, by injection, or even through implantable medical devices, as the final degradation products comprise of sole l-malates. These products can be further utilized in the TCA cycle and metabolized into CO_2_ and water, constituting no secondary harm to the body or no residual deposition in the body.

However, after decades of PMLA research and technological development, challenges still remain. The first problem lies in the PMLA’s unscalable production and functionalization for medicinal purposes. Following current manufacturing flow and purification processes, the regular yield of bioproduced PMLA remains on a scale of grams per batch, taking up to two weeks per production cycle [59]. Further functionalization that readies PMLA-based compounds for biological use, especially through conjugation chemistry, only produces the final product on a scale of milligrams after purification steps; moreover, each purification step may require different solvents, apparatuses, reaction parameters, etc., all leading to complications in scalability from bench to production plant, let alone from clinical trial to bedside [60].

The second challenge remains that the genes responsible for PMLA production in several microorganisms are yet to be fully uncovered. Once identified, these genes could be introduced into the commonly used industrial microbes for the biosynthesis of this precious natural product using metabolic engineering, thereby largely lowering the manufacture cost, boosting the yield, and commercializing the product. For this reason, the *M*_w_ of PMLA cannot be controlled during its biosynthesis, so it is impossible to tailor the length of bioproduced PMLA as desired. In parallel, the relationship between the *M*_w_ of PMLA and its suitability as a delivery platform also needs to be determined. The higher the *M*_w_ of PMLA, the greater its loading capacity and the longer its delivery time is before its hydrolytic degradation; however, this relationship has not been quantitatively defined or precisely assessed.

Last but not least, there are unexplored opportunities in which PMLA could be used for more applications other than a delivery platform. With unrivaled hydrophilicity, PMLA-based drug delivery can be applied to many fields insofar uncharted, such as kidney diseases and urological cancers/infections. Besides, as a fully degradable biopolymer, PMLA could be useful in fields other than biomedical research, such as the food or pharmaceutical industry for external packaging. Its applicability to these fields would only help promote research into its medicinal development.

In closing, PMLA is currently one of remarkable materials for many biomedical applications, including pharmaceutical delivery and tissue engineering. Its nontoxic, non-immunogenic, and fully degradable features make it a perfect medicinal platform, holding great potential in targeted biomolecular delivery and many other pharmaceutical and biological applications.

## Data Availability

Not applicable.

## Abbreviations

*A. melanogenum*: *Aureobasidium melanogenum*
*A. pullulans*: *Aureobasidium pullulans*
acetyl-CoA: Acetyl coenzyme A
AMP: Adenosine monophosphate
AON: Antisense oligonucleotide
AP-2: Angiopep-2
ATMA: Alkyltrimethylammonium
ATP: Adenosine triphosphate
BBB: Blood–brain barrier
BBTB: Blood–brain tumor barrier
bFGF: Basic fibroblast growth factor
Ca^2+^: Calcium ion
CaCO_3_: Calcium carbonate
CO_2_: Carbon dioxide
-COOH: Carboxyl
CTLA-4: Cytotoxic T-lymphocyte–associated antigen 4
CTX: Cyclophosphamide
CuSO_4_: Copper sulfate
Cys-CS: Cysteine-conjugated chitosan
DMMA: Dimethylmaleic anhydride
DNA: Deoxyribonucleic acid
DOX: Doxorubicin
*E.coli*: *Escherichia coli*
EGFR: Epidermal growth factor receptor
Gat1: Gamma-aminobutyric acid transporter 1
GBM: Glioblastoma
Gd-DOTA: Gadolinium-DOTA
*H. pylori*: *Helicobacter pylori*
HER2: Human epidermal growth factor receptor 2
i.v.: Intravenous
ICG: Indocyanine green
K_2_CO_3_: Potassium carbonate
LLL: Trileucine
malyl-CoA: Malyl coenzyme A
mAb: Monoclonal antibody
MRI: Magnetic resonance imaging
mRNA: Messenger RNA
Ms: Methylsulfonyl
*M*_*W*_: Molecular weight
Na_2_CO_3_: Sodium carbonate
NADH: Nicotinamide adenine dinucleotide
NIR: Near-infrared
NRPS: Nonribosomal peptide synthetase
-OH: Oxhydryl
*P. polycephalum*: *Physarum polycephalum*
PD-1: Programmed cell death 1
PEG: Polyethylene glycol
PEI: Polyethyleneimine
PMLA: Poly(β-L-malic acid)
rh: Rhodamine
SA: Succinic anhydride
TAT: Transactivator of transcription
TCA: Tricarboxylic acid
TfR: Transferrin receptor
w/v: Weight/volume
ZnO: Zinc oxide
